# An exploratory case study investigating the implementation of a novel knowledge translation strategy in a pandemic: the Pandemic Practice Champion

**DOI:** 10.1186/s43058-022-00294-2

**Published:** 2022-04-18

**Authors:** Bonnie Stevens, Mariana Bueno, Megha Rao, Christabelle Almeida, Anna Cotic, Laurie Streitenberger, Bonnie Fleming-Carroll, Karen Breen-Reid

**Affiliations:** 1 Lawrence S. Bloomberg Faculty of Nursing & Faculties of Medicine and Dentistry, University of Toronto, 155 College Street, M5T 1P8 Toronto, Canada; 2 Child Health and Evaluative Sciences, The Hospital for Sick Children, Peter Gilgan Centre for Research and Learning (PGCRL), 686 Bay Street, 6th floor, M5G 0A4 Toronto, Canada; 3 School of Kinesiology, The University of Western Ontario, 1151 Richmond St, N6A 5B9 London, Canada; 4 The Hospital for Sick Children, 555 University Ave, M5G 1X8 Toronto, Canada

**Keywords:** Champions, Stakeholder, Context, Use of evidence, Implementation strategy

## Abstract

**Background:**

The clinical Pandemic Practice Champion (PPC) role was created in a large tertiary pediatric hospital as a knowledge translation (KT) strategy for implementing COVID-19 evidence-based knowledge. We aimed to describe the core components of the PPC role, the process of implementing the role, and the factors that hindered or facilitated role implementation.

**Methods:**

An exploratory case study was undertaken. Semi-structured interviews were conducted virtually with stakeholders including PPC, managers, and front-line health care professionals (HCP). A directed approach to qualitative content analysis consistent with the Consolidated Framework for Implementation Research (CFIR) guided the analytic process. Inductive analyses and three stages of thematic synthesis were also conducted.

**Results:**

Four PPC, 3 managers, and 6 HCP were interviewed. The core components of the PPC role consisted of (a) acting as knowledge experts and educators, (b) problem-solving for complex patient care issues, (c) conducting crisis management, and (d) acting as a resource to management, HCP, and families. *Facilitators* for successful implementation included access to external information, a supportive organizational context and culture, dedicated time and resources, and leadership support. Lack of clarity of role definition, insufficient time, pandemic uncertainty and fatigue, inability to change infrastructure, and access to external information hindered implementation.

**Conclusion:**

The PPC role was successfully implemented within a crisis context. Key barriers (role clarity, time, resources) and facilitators (organizational and leadership support) need to be considered when implementing the PPC role in practice. Future studies are needed to determine the intervention effectiveness of the champion role in changing HCP behavior and health outcomes and further examine implementation processes and mechanisms.

Contributions to the literature

- We contributed to the implementation science literature by qualitatively exploring the Pandemic Practice Champion (PPC) role as an implementation strategy in a large pediatric acute healthcare institution during the pandemic.
- From qualitative interviews with multiple stakeholders, we learned that the PPC role consisted of (a) acting as knowledge experts and educators, (b) problem solving for complex patient care issues, (c) conducting crisis management, and (d) acting as a resource to management, HCP, and families. *Facilitators* for successful implementation included access to external information, a supportive organizational context and culture, dedicated time and resources, and leadership support. *Barriers* included uncertainty on role definition, insufficient time, pandemic uncertainty and fatigue, inability to change infrastructure, and access to and influence of external information.
- Results will standardize and inform the role of the clinical champion for effective implementation across a wider range of healthcare settings.

## Background

Following the declaration of the COVID-19 pandemic by the World Health Organization (WHO), the Hospital for Sick Children (SickKids) developed the Pandemic Practice Champion (PPC) role as a knowledge translation (KT) or implementation strategy to address the rapidly changing knowledge needs of Health Care Professionals (HCP) and to ensure quality care for children and their families. One of the challenges in implementation science is the inconsistent use of terminology and inadequate definition of strategies. For example, while the term “implementation” is used broadly in the USA, in Canada, implementation is frequently referred to as KT.

Clinical champions notably have intrinsic interest and commitment to implementing change; are enthusiastic, dynamic, and persistent; and have the strength of conviction [1]. Powell et al. (2015), in his compilation of 73 implementation strategies, identified champions as an implementation strategy and defined them as “individuals who dedicate themselves to supporting, marketing, and driving through an implementation, overcoming indifference or resistance that the intervention may provoke in an organization” [2].

Flanagan and colleagues (2018) [3], in a study of 38 stroke units in the USA, reported that clinical champions implemented and co-ordinated care processes, built collaborations, educated colleagues, problem solved, monitored progress, and standardized care. Similarly, Soo, Berta, & Baker (2009) [4] reported that the roles of clinical champions encompassed education, advocacy, relationship building, and boundary management. Although clinical champions have been highly promoted for integrating knowledge into practice, there is little empirical evidence on how to effectively operationalize the role in an acute care hospital within a pandemic context. Therefore, our aims were to describe (a) the core components of the PPC role, (b) the processes of implementing the role, and (c) factors that hindered or facilitated the implementation of the role in a pediatric hospital setting.

### Theoretical framework

The Consolidated Framework for Implementation Research (CFIR) [5] was used as the study’s conceptual basis. The CFIR is a determinant framework that focuses on five domains of implementation—the innovation, implementor, implementation process, and facilitators and barriers in the inner and outer setting, thus aligning well with the aims of this study.

## Methods

### Study design, setting, and sample

An exploratory case study design was undertaken in one large tertiary pediatric health institution in Canada. Eligible participants included (a) PPCs who were in the role for a minimum of 8 weeks and (b) HCPs and managers identified by the PPC who were familiar with their role. The list of PPCs was provided by the Associate Chief of Nursing Education, and eligible individuals were invited via email to participate in the study. Once they agreed to participate, the PPCs were introduced to the researcher and asked to identify a manager and staff nurse who was familiar with the role. We also invited these individuals by email to participate. We aimed to recruit and interview 4–5 individuals from each of the three key stakeholder groups for a total sample size of 12–15 participants. A limitation to this case study is that it only includes qualitative data. However, we were able to conduct interviews from three different groups of stakeholders to get a broader perspective and deeper understanding of the PPC role.

### Data collection methods

Semi-structured individual interviews were conducted virtually. Quality Improvement (QI) Committee approval at the hospital was obtained prior to study commencement (deemed appropriate by the Research Ethics Board for QI interventions). Those who agreed to participate were approached using a secure online platform or by telephone, and electronic consent forms were securely transmitted. Sixty-minute interviews were conducted by a trained interviewer and audio-recorded, with permission. The interview guide was adapted from the CFIR Guide (https://cfirguide.org/tools). The interview guide was finalized by study team members to ensure consistency with the study aims and reviewed by an expert on the PPC role. The interview guide was then piloted with one PPC and was accepted with no changes required. Demographic variables were recorded for sample description.

### Data management and analyses

Interviews were uploaded on a hospital password-protected computer, encrypted, and sent to a professional transcriber for de-identification and transcription.

A directed (deductive) approach [6] to qualitative content analysis, consistent with a priori themes based on the study aims and CFIR, guided the analytic process; inductive analyses beyond the CFIR-based questions were also included. Thematic syntheses were conducted by (1) coding of transcribed interviews line-by-line by three individuals [7], (2) development of descriptive themes related to the study aims and guided by CFIR, and (3) synthesis of descriptive themes through discussion. We identified determinants across groups of stakeholders. Methodological rigor was maintained through constant comparison, reflexive analysis, and debriefing amongst analysts; new codes were added as they emerged from debriefing sessions.

## Results

### Demographic characteristics

A total of 13 participants were included in the study: 4 PPCs, 3 managers, and 6 HCP. PPCs were primarily senior nurses, some with educational experience, within the hospital setting. Characteristics of all participants are reported in Table 1. This sample size was determined to be adequate for content saturation and the proposed qualitative analyses as no new codes or themes emerged [8].

**Table 1:** Demographic Characteristics of Study Participants

Participant	PPC^a^ *n* =4	Manager *n* =3	HCP^b^ *n* =6
	*n* (%)	*n* (%)	*n* (%)
Gender			
Female	4(30.8)	3(23.1)	5(38.5)
Male	0(0.0)	0(0.0)	1(7.7)
Education			
Masters	3(23.1)	3(23.1)	4(30.8)
Bachelors	1(7.7)	0(0.0)	2(15.4)
Years of experience			
<1	0(0.0)	1(7.7)	0(0.0)
1 to 5	2(15.4)	1(7.7)	3(23.1)
6 to 10	2(15.4)	0(0.0)	2(15.4)
>10	0(0.0)	1(7.7)	1(7.7)

^a^*PPC* pandemic practice champion, ^b^*HCP* health care professional

### Stakeholder perceptions of PPC role

Perceptions of the PPC role from the PPC, managers, and HCP are captured in Table 2. The results are integrated across the stakeholder groups by the overarching theme and subthemes guided by the five domains of the CFIR (Intervention, Process, Inner Setting, Outer Setting, Individuals). Thirty-two descriptive themes were identified across the CFIR domains. There were 8 descriptive themes identified by all three groups of stakeholders, 18 identified by two groups, and 30 identified by only one group. Factors that were most consistently recognized fell predominantly within the Process and Inner Setting CFIR domains. As the CFIR themes are interactional, contingent and reciprocal relationships between themes are highlighted where possible.

**Table 2:** Components of the PPC Role: Domains and constructs identified across three stakeholder groups

CFIR Domain	Construct within Domain	Constructs and Examples	PPC	Manager	HCP
Characteristics of the Intervention	Complexity/Adaptability (include CFIR definition) -	Complexity: Managers described the PPC as knowledge experts who addressed the complexities of providing clinical care during Covid and implementing a multitude of rapidly changing evidence.		✓	✓
		Adaptability and Available for Crisis Management**:** Managers described the necessity for the PPC to be able to adapt quickly and use problem solving skills to determine what worked and where they could leverage opportunities.		✓	✓
		HCP considered PPC as crisis managers.		✓	✓
	Relative advantage	Relative Advantage: PPC viewed their role as that of a clinical champion, they were subject experts on Covid as well as experts in implementation strategies. This role had a relative advantage over more traditional nurse educators.	✓	✓	
	Evidence Strength and quality	Validity of Evidence: HCP considered the PPC as a resource for staff. They verified quickly changing evidence and enforced information between managers, clinical units and with families.	✓		✓
	Cost	Value Added: Manager: The PPC were well received by the staff. They provided real time Covid education for HCP. Although the PPC role did not equate to cost savings they were cost effective.		✓	

### Overarching theme: successful implementation of the PPC role

The PPC was a successful implementation strategy within the era of the pandemic in an acute care setting. One PPC stated “This was an amazing opportunity. I really enjoyed being a key resource for frontline clinicians during this time and… I really enjoyed working on the quality improvement and education side of things” (005, p.24)

### Subtheme #1—core components of the PPC intervention

Our findings from key stakeholders indicated that the PPC intervention consisted of (a) acting as knowledge experts and educators, (b) problem-solving for complex patient care issues, (c) conducting crisis management, and (d) acting as a resource to management, HCP, and families (Table 2).

### Subtheme #2—process of implementing the PPC intervention

The implementation process involved engaging, planning, executing, and evaluating the PPC role as a KT strategy. There was consistency between the three groups of stakeholders in the individual implementation processes (Table 3).

**Table 3:** The process of implementing the PPC role

CFIR Domain	Construct within Domain	Constructs and Examples	PPC	Manager	HCP
Implementation Process	Engaging	Champions: HCP: Subject experts/key knowledge stakeholders. Champions at the local level for translating information, policies into practice.	✓	✓	✓
		Opinion Leaders: PPC: Providing support. Engaging with staff and building relationships and collaborations.		✓	
		Manager: Connecting the team and key stakeholders.		✓	
	Planning	Training: Manager: Educators who received adequate preparation for the PPC role. Some considered there was insufficient development of PPC role by Nursing Education Executive.		✓	
	Executing	Communicators/Champions: HCP: Centralized source of information, consolidating information, confirming, clarifying, asking questions. Liaising/ linking with staff. Supporting staff through collaboration.	✓		✓
		PPC: Responding in real time. Troubleshooting and problem solving Conflict management. Acting as a resource.	✓	✓	
		Manager: Synthesizing knowledge.	✓	✓	✓
	Reflecting & Evaluating	Evaluating and Monitoring Outcomes: PPC: Evaluating guidelines and policy implications, monitoring educational processes and standardizing care.	✓		
		Manager: Evaluating information needs.		✓	

*Engaging* involved collaboration with staff and building relationships. PPCs were viewed as local champions who were subject experts or key knowledge stakeholders that provided support for practice change. Managers saw the PPC engaging with the key stakeholders by being “connectors from the frontline to the clinical manager” Manager 004, p.7

*Planning* included identifying of stakeholders’ needs, the gaps in communication, and knowledge integration for frontline staff. The PPC received training for the PPC role. They co-designed and implemented the PPC role integrating adult learning principles, KT, and empathy to create meaningful relationships and a caring and supportive environment.

*Executing* the intervention with fidelity was iterative with the qualities of the individual and the characteristics of the intervention. Nurses relied on the PPC to clarify, confirm, and synthesize information in real time or be a sounding board for asking questions and listening.

“They synthesized information and just got down to what people’s key questions were to alleviate anxiety and help them go about their day – that was critical” Manager 004, p.7

*Evaluating* included monitoring educational processes, standardizing care, and determining the quality of the implementation process. Stakeholders considered different processes and implications for evaluating guidelines and policy implementation through the lens of the PPC role. However, given the newness of the role, they often did not recognize success.

“We didn’t know what we didn’t know.... and I think being open and flexible and being aware where more information or gaps needed to be filled was super important” Manager 004, p.17

### Subtheme #3—barriers and facilitators to PPC role implementation

There was a high level of consistency between stakeholder groups on what hindered (barriers) or facilitated (facilitators) the implementation of the PPC role (Table 4).

**Table 4:** Identification of Facilitators and Hindrances for the Implementation of the PPC Role

CFIR Domain	Construct within Domain	Constructs and their Explanation	PPC	Manager	HCP
Outer Setting	Patient Needs & Resources	Facilitators: HCP: Supportive to patient and patient’s family needs. Indirectly impact the experience of patients and families.			✓
		Hindrances: HCP: Lack of sustainable resources.			✓
	External Policies and Incentives	Facilitators: HCP: Policies from external agencies – e.g., Ministry of Health regarding the pandemic.	✓		✓
		Hindrances: HCP: Insufficient resources/ detail from province.			✓
		PPC: Lack of synchronization of information and timing, and fear of different information from news agencies in the pandemic.	✓		
	Cosmopolitanism	Facilitator: HCP: External benchmarking done at hospital.			✓
Inner Setting	Structural Characteristics	Facilitator: HCP: Central office.		✓	✓
		Hindrances: HCP: Lack of role clarity/awareness/recognition/ introduction to role. Unknown as to accountability in the organization structure.	✓		
		Manager: Challenges adapting to multiple settings within the organization	✓		✓
	Availability of Resources	Facilitators: HCP: Voicera phones	✓	✓	✓
		HCP: Educational/ Training Sessions.	✓		✓
		PPC: Educational materials/ binders/ handouts.	✓		
		HCP: Email Communications/ local telephone.		✓	✓
		Covid website. PPC: Data base for questions.	✓		✓
		HCP: Signage.	✓		✓
	Networks & Communications	Facilitators: HCP: Informal communication. Centralized source of information.			✓
		Consolidating information. Confirming, clarifying, asking questioning			✓
		Communicating with staff during Huddles/ Rounds.	✓		
		PPC: Access to key people.	✓		
		Manager: Disseminating information.		✓	
		Hindrances: Manager: Variability in communication styles.		✓	
		HCP: Inconsistency in communication for HCP. Information overload.			✓
		Facilitator and Hindrance: HCP: Training available but insufficient for formal PPC role.		✓	
	Culture	Facilitators: HCP: Culture of Openness to Change.			✓
		Supportive unit culture.	✓	✓	✓
		PPC: Staff are open to change, culture of safety, earning.	✓		
		Hindrances: HCP: Acute care culture that lacked adaptability.			✓
		Facilitators: HCP: Culture of Excellence.			﻿✓
		PPC: Amazing work that trickles through the organization.	✓		
		Manager: Impact on practice change, organizational responsiveness, innovation, and adaptability.	✓	✓	✓
	Implementation Climate	Hindrances: HCP: Tension for change.			✓
		PPC: Poor attitude towards change.	✓		
		Manager: Contention and disagreement on how to move forward.		✓	
		Hindrances: HCP: Compatibility.			✓
		Lack of buy-in from some staff in some areas, hostile responses at times.	✓		✓
		Facilitators: PPC: Overall, PPC well received.	✓		
		MG: Compatible with roles and initiatives.	✓	✓	
		Hindrances: HCP: Relative Priority.			✓
		Manager: Competing priorities with care needs.	✓	✓	✓
		HCP: Organizational Incentives and Rewards.			✓
		Facilitators: HCP: Learning climate.			✓
		Openness to learning and implementation.	✓	✓	✓
		Hindrances: HCP: Some units more receptive than others.	✓	✓	✓
		HCP: Time.	✓	✓	✓
		PPC: Inability to connect with people resources in timely manner.	✓		
		Manager: Insufficient time to getting the word out.		✓	
		HCP: Pandemic fatigue and unfamiliarity.			✓
		Facilitators: HCP: Goals and Feedback.			✓
		MG: Similar goals to other roles and initiatives.		✓	
	Readiness for Implementation	Facilitators: HCP: Leadership Engagement/ Support	✓		
		PPC: Leadership mentors available.		✓	
		MG: Leadership involvement with PPC role development.	✓		✓
		Ready for the PPC role.		✓	
		HCP: Access to Knowledge and Information.			✓

*Barriers* to the successful implementation of the PPC role included uncertainty on role definition, insufficient time, pandemic uncertainty and fatigue, inability to change infrastructure, and access to and influence of external information. There was uncertainty about the PPC role definition amongst stakeholders. While some felt that the PPC role was primarily targeting HCP, others considered that the role positively affected the experiences of patients and families.

Insufficient time was commonly mentioned as a barrier, especially insufficient time to “get the word out” about constantly changing data, which was not always readily accessible. Also, the need for information and readiness to receive it were not always simultaneous:

“The need for information was high...but burnout was an issue, morale was low, people were scared … but at the same time they were looking for information … and that was a huge facilitator” HCP 008, p.17

Pandemic fatigue and uncertainty were also barriers; often palpable yet unfamiliar. Ultimately, the novelty and pressure of the situation came through strongly.

“It’s a pandemic – nobody knows what to do and it’s all brand new. And none of us have lived through this before” HCP 009, p.26

People were overwhelmed with the overabundance of information and stress. When fatigued, people did not always want to hear what the new changes were and responses were sometimes varied, hostile, or unappreciated; attributed to competing priories with care needs on the unit.

*Facilitators* included access to external information, a supportive organizational context and culture, dedicated time and resources, and leadership support. Availability of external information, policies, and guidelines were considered facilitators to implementing the PPC role when available and barriers when they were not. The PPC looked to other organizations for guidance, on what they were doing and what worked well for them to ultimately guide procedure and policy development and benchmark their practices.

There was consistent recognition between stakeholders on the influence of organizational contextual factors (or the impact of the inner setting domain described in CFIR, Table 4). The physical and social architecture of the organization was thought to facilitate the implementation process. The PPC had a central office to meet as well as dedicated time and KT strategies and resources. They used huddles, rounds, mini educational strategies, phones, the hospital-wide COVID-19 website, and signage to mobilize new knowledge. Time allowed them to engage in communication that embodied clarifying, consolidating, confirming, and disseminating information.

“They’re acting like a bridge for knowledge transfer. Because there is so much information that’s out there and practice is changing, and information is changing... I just feel like its’s been a really good role to promote and disseminate information” Manager 004, p.4

However, they also faced barriers on communication, including varied communication styles and patterns and varied ways that information was received and processed.

Organizational culture embraced the norms, values, and basic assumptions of the collective. Multiple cultures co-existed including the cultures of excellence, change, and unit/organizational culture which facilitated the PPC role implementation. Within the organizational culture, the capacity for change, or the extent to which the intervention was accepted, valued, and rewarded was important. The PPC was generally well received, but the tension for change or individual poor attitudes towards change, competing priorities, and disagreement on moving forward occasionally threatened to hinder practice change. The PPC also described their lack of recognition and accountability in the organization structure.

Leadership support was an essential facilitator for implementation success. Leaders provided mentorship, feedback, and crisis management in the implementation process. They also suggested that it was their responsibility to make a case for sustaining the role and articulating the return on investment from a staff and patient safety perspective.

## Discussion

Identifying and supporting champions such as the PPC can play an important role in a successful implementation strategy (Powell et al., 2015) [2]. Through exploring the perspectives of various stakeholders, we increased our understanding of the PPC role, the process of how it was implemented and the factors that facilitated or hindered its’ success.

### The role

Research to date has concentrated more on strategies used for practice change than on the personal characteristics of the practice champions. In this study, the PPC was primarily experienced nurses who had had a keen interest in education and quality improvement. They often possessed leadership experience, were excellent communicators, and had a passion for assisting their colleagues. They were educators who acted as resources to clarify and re-enforce new evidence; support key relationships; engage in troubleshooting, problem-solving, and resolving crises; and evaluate guidelines and policies in light of new information. The role of clinical champions was also the topic of a systematic review by Wood et al. (2020) [9] in the mental health field. From 13 studies, they concluded that clinical champions assisted with the more expedient implementation of novel interventions by overcoming systems barriers and enhancing staff engagement and motivation. Demes et al. (2020) [10] identified role components including communication and persuasion, proactivity, horizontal and collective leadership, sense of responsibility and accountability, dedication and motivation, and ability to inspire and motivate and encourage learning as key characteristics of a successful champion in quality health initiatives in Haiti. Bonawitz et al. (2020) [11], also using the CFIR framework, articulated that influence, ownership, physical presence at the point of change, persuasiveness, grit, and leadership style were attributes that bode well for effective practice change in inpatient postpartum contraceptive care. The findings of these studies relate to the PPC role; although the sites and settings vary, the champions’ core skills and abilities for communication, relationship-building, persuasiveness, leadership, vision, and passion for learning are salient across all fields.

### Process for implementing the champion role

To successfully implement new knowledge, the PPC was either required to build new relationships or fortify pre-existing ones to persuade and enable others to adopt a change and gain support from their managers and peers. This change was accomplished by their ability to communicate effectively and clarify the educational needs of others [3]. To simply put it, they connected individuals and information by consolidating, confirming, and clarifying evidence. They were nimble to respond, troubleshoot, and address crises. Due to the complex and dynamic needs, PPC focused on simple tactics allowing multiple iterations of testing to fail, while understanding what elements worked best [12]. The PPC provided a model for integrated KT [13] where they engaged knowledge users in a collaborative process. In this co-production of knowledge, it is hypothesized that synergies derived from the collaboration will result in better science, more relevant and actionable research findings, increased use of the findings in policy or practice, and mutual learning.

Factors from the CFIR inner setting or hospital/unit culture were key in the process of successful implementation of the PPC role. Various cultures including cultures for change, and excellence, and an implementation climate that embraced tension for change were required.

Their perspectives of the PPC on the implementation process were highly valuable to researchers and will help to standardize the practice champion role and inform the development of a formal job description, and an account of how and where they best work. New knowledge will inform future exploration determining their implementation and intervention effectiveness locally and potential adoption elsewhere. An important next step is to study relationships and mechanisms underlying KT strategies [14].

### Barriers and facilitators

Information overload, pandemic fatigue and uncertainty, and insufficient time were identified as barriers. These factors were not experienced in isolation but captured within setting and process factors in the CFIR. The PPC addressed these challenges by supporting, marketing, and driving through an implementation, and overcoming indifference, resistance, or setbacks that the intervention may provoke in an organization [2, 3]. Lack of role clarity was also identified barrier to implementing the PPC role. This is not unanticipated and even considered strategic given a broad role description that permitted the evolution of the role as it emerged. The availability of external information, physical space, dedicated time and resources, a positive organizational context, ability to network and communicate, buy-in, and leadership support was all noted to facilitate the implementation of new knowledge.

### Study limitations

Several limitations were evident in this study. Given the timing of this study during a pandemic crisis period, this study was limited to a single-center exploratory case study. The PPC role is new and unique and therefore has limited evidence of support within the literature. While a strong qualitative methodology for analyzing the data was employed, the small sample size and 1-h virtual interview were limited to the convenience sample of PPC, managers, and staff nurses who made themselves available.

## Conclusion

The COVID-19 pandemic has produced the “silver lining” of inspiring healthcare innovation [12]. Practice champions offer the potential for practice change success; however, champions alone may not be successful [1]. Evidence is still emerging around innovative roles of practice champions such as the PPC and determinants that affect their implementation. Successfully implementing the PPC role relies on accessing the most recent evidence-based knowledge, conducting flexible education and educational outreach strategies, engaging in effective and timely communication, and experiencing a supportive unit culture and leadership buy-in. Further clarification of the PPC role, addressing the adaptation of the role in multiple settings, compatibility and time, and accountability requirements, is recommended. Future studies are needed to progress from the description of factors as outlined in the CFIR framework that determines the implementation success of KT strategies to a more in-depth examination of implementation processes, mechanisms, and intervention effectiveness by which change and outcomes occur within complex systems, such as healthcare.

## Data Availability

The datasets used and/or analyzed during the current study are available from the corresponding author on reasonable request.

## Abbreviations

CFIR: Consolidated Framework for Implementation Research
HCP: Health care professionals
KT: Knowledge translation
PPC: Pandemic Practice Champion
WHO: World Health Organization
